# Leukemia Stem Cell-Released Microvesicles Promote the Survival and Migration of Myeloid Leukemia Cells and These Effects Can Be Inhibited by MicroRNA34a Overexpression

**DOI:** 10.1155/2016/9313425

**Published:** 2016-04-05

**Authors:** Yue Wang, Qian Cheng, Jing Liu, Min Dong

**Affiliations:** ^1^Department of Hematology, The Affiliated Hospital of Guilin Medical College, Guilin, Guangxi 541001, China; ^2^Department of Hematology, The Third Xiangya Hospital of Central South University, Changsha 410013, China

## Abstract

Leukemia stem cells (LSCs) play the major role in relapse of acute myeloid leukemia (AML). Recent evidence indicates that microvesicles (MVs) released from cancer stem cells can promote tumor growth and invasion. In this study, we investigated whether LSCs-released MVs (LMVs) can regulate the malignance of AML cells and whether overexpression of tumor suppressive microRNA (miR), miR34a, is able to interrupt this process. LSCs were transfected with miRNA control (miRCtrl) or miR34a mimic for producing LMVs, respectively, defined as LMVs^miRCtrl^ and LMVs^miR34a^. The effect of miR34a transfection on LSC proliferation and the effects of LMVs^miRCtrl^ or LMVs^miR34a^ on the proliferation, migration, and apoptosis of AML cells (after LSC depletion) were determined. The levels of miR34a targets, caspase-3 and T cell immunoglobulin mucin-3 (Tim-3), were analyzed. Results showed that (1) LMVs^miRCtrl^ promoted proliferation and migration and inhibited apoptosis of AML cells, which were associated with miR34a deficit; (2) transfection of miR34a mimic inhibited LSC proliferation and increased miR34a level in LMVs^miR34a^; (3) LMVs^miR34a^ produced opposite effects as compared with LMVs^miRCtrl^, which were associated with the changes of caspase-3 and Tim-3 levels. In summary, LMVs support AML cell malignance and modulating miR34a could offer a new approach for the management of AML.

## 1. Introduction

Acute myeloid leukemia (AML) is an aggressive disease characterized by rapid proliferation of immature myeloid cells in the bone marrow resulting in dysfunctional hematopoiesis [1]. Although current treatments can induce remission, many AML patients end in relapse owing to the presence of dormant leukemia stem cells (LSCs) which are resistant to chemotherapies. LSCs are able to self-renew, proliferate, and differentiate extensively, which are crucial for the initiation and maintenance of AML. Cancer stem cells are known to affect cancer progression in part through secretory factors [2]. Microvesicles (MVs) are membrane vesicles that are released from cells undergoing stress, activation, or apoptosis, which can mediate cell-to-cell communication by transferring proteins, mRNAs, and microRNAs (miRs) and lipids between cells [3, 4]. Recent evidence has shown that MVs secreted from cancer stem cells could interact with surrounding cancer cells and stromal cells, which may be involved in tumor progression and metastasis [5, 6]. For example, gastric cancer stem cell-secreted MVs were shown to promote gastric cancer cell proliferation and migration [5]. Moreover, MVs secreted from renal cancer stem cells induced in vitro and in vivo angiogenesis and favored lung metastasis [6]. However, it is unknown whether and how LSCs-derived MVs (LMVs) regulate the malignance of AML.

miRs are small noncoding RNAs that function as major players of posttranscriptional gene regulation within diverse cell types. Dysregulation of tumor-suppressive miR34a has been implicated in AML [7]. Forced expression of miR34a resulted in granulocytic differentiation of AML blasts, suggesting that increasing miR34a levels might become therapeutically useful for AML [8]. In addition, several miR34a downstream target genes were identified in AML. For example, caspase-3 was reported as an essential molecule for programmed cell death, differentiation, and survival of AML [9, 10]. T cell immunoglobulin mucin-3 (Tim-3) was highly expressed in human AML cells, which could trigger prosurvival and proinvasive signaling [11, 12]. Since MVs may act as paracrine or endocrine mediators by a horizontal transfer of genetic information into the recipient cells [13], we proposed that overexpression of miR34a in LSCs could synchronously increase the level of miR34a in LMVs, which could be able to abrogate LMVs-induced effects on AML.

In this study, we aimed to investigate the effects of LMVs on the proliferation, migration, and apoptosis of AML cells. The miR34a associated caspase-3 and Tim-3 pathways in LMVs-mediated effects were also investigated.

## 2. Materials and Methods

### 2.1. Cell Culture and Sorting

Human AML cell line KG1a (ATCC, Manassas, VA) was maintained in suspension culture with Dulbecco's Modified Eagle's medium (DMEM) supplemented with 10% fetal bovine serum (FBS) and 100 U/mL of penicillin and streptomycin. Since LSCs were defined as CD34+CD38− fraction of KG1a cells and all of KG1a cells were CD34+, LSCs were enriched by indirectly labeling with CD38-FITC antibody and anti-FITC microbeads according to the manufacturer's instructions (Miltenyi Biotec, Bergisch Gladbach, Germany). Briefly, cell suspensions were centrifuged at 125 g for 10 min and washed in phosphate-buffered saline (PBS). Then, cell pellets were resuspended in separating buffer (PBS containing 0.5% bovine serum albumin) and incubated with CD38-FITC antibody for 30 min. After washing with PBS, cell pellets were resuspended in separating buffer containing Anti-FITC MicroBeads for 15 min. After several washing steps, filtrates (CD34+CD38− cells) were collected by a LD column using a MidiMACS separator system. Throughout the sorting procedure, cells were kept at 4°C and analyzed immediately by flow cytometry. The remaining cells after depletion of LSCs were defined as AML cells, which were used for coculture studies.

### 2.2. Flow Cytometry Analysis

1 × 10^5^ cells were resuspended in PBS and incubated with antibodies against LSC surface antigens including CD34 and CD38 for 30 min at 4°C. The IgG isotype was served as negative control. LSCs were used to generate LMVs for coculture with AML cells.

### 2.3. Cell Transfection

5 × 10^5^ cell suspensions were seeded in 6-well plates and transfected with miR34a mimic (Sigma, 100 nM) or miR control (miRCtrl; Sigma, 100 nM) using Lipofectamine 2000 transfection reagent (Invitrogen, CA) for 2 days. Transfection efficacy was examined by Real-Time Quantitative Reverse Transcription PCR (qRT-PCR).

### 2.4. qRT-PCR

Total RNA was extracted with TRIzol Reagent (Invitrogen) according to the manufacturer's instructions. cDNA was synthesized using miScript reverse transcription kit (Qiagen). Quantitative real-time PCR was conducted with miR34a specific primers and miScript SYBR Green PCR Kit (QIAGEN) on a real-time PCR system (Bio-Rad). Small nuclear RNA U6 was used as an internal control. All experiments were carried out in triplicate. Relative expression of miR34 was calculated using the 2^−ΔΔCT^ method [14].

### 2.5. Isolation and Identification of LMVs^miRCtrl^ and LMVs^miR34a^

MVs generated from LSCs transfected with miRCtrl or miR34a mimic were, respectively, defined as LMVs^miRCtrl^ and LMVs^miR34a^. MVs were collected from cell culture medium and purified by differential centrifugations as previously reported [15]. The particle number and size distribution of secreted MVs were measured by NanoSight NS300 instrument (Malvern Instruments, Amesbury, UK) according to the manufacturer's instructions [16].

### 2.6. Coculture of AML Cells with Vehicle, LMVs^miRCtrl^, or LMVs^miR34a^

Isolated LMVs^miRCtrl^ or LMVs^miR34a^ (50 *μ*g) were resuspended with fresh culture medium. AML cells were, respectively, treated with vehicle (veh, fresh culture medium), LMVs^miRCtrl^, and LMVs^miR34a^ for 24 hrs in an incubator (37°C, 5% CO_2_). AML cells were collected and subjected to cell apoptosis, proliferation, and migration assays.

### 2.7. Cell Apoptosis Assay

The terminal deoxynucleotidyl transferase-mediated dUTP-digoxigenin nick end labeling (TUNEL) assay was performed according to the manufacturer's instructions (Boehringer Mannheim, Germany). In brief, cells were fixed with 4% paraformaldehyde for 30 min and subsequently permeabilized with 0.1% Triton X-100 in 0.1% sodium citrate for 2 min. The cells were incubated with 50 *μ*L TUNEL reaction mixture for 1 h at 37°C. Labeled cells were analyzed by flow cytometry.

### 2.8. Cell Proliferation Assay

Cells were seeded in 96-well plate and added with 10 *μ*L methyl thiazolyl tetrazolium (MTT, 12 mM; Invitrogen, NY) solution. After 4 hrs of incubation, dimethyl sulfoxide- (DMSO-) isopropanol (1 : 1) solvent was added directly to the cell suspension and incubated for 30 min. The absorbance was measured at 570 nm.

### 2.9. Cell Migration Assay

Cells were suspended in serum-free medium and added to the top chambers of 24-well transwell plates (Corning Inc., NY). The lower chambers were filled with 500 *μ*L complete culture media. Transwell plates were incubated for 24 hrs in a 37°C cell incubator. Migrated cells were fixed with 4% paraformaldehyde, stained with 0.1% crystal violet, and enumerated in ten randomly selected fields under a light microscope.

### 2.10. Western Blot

Proteins were extracted from cells with lysis buffer containing protease inhibitor (Roche Diagnostics). Proteins were separated by SDS-PAGE and transferred onto PVDF membrane. The PVDF membrane was blocked with 5% nonfat milk in 1x Tris-buffered saline with Tween-20 (TBST, pH 7.6) at room temperature (RT) for 1 h and then incubated with primary antibody against Tim-3 (1 : 200; Santa Cruz) at 4°C overnight. *β*-actin (1 : 40000; Sigma) was used to normalize protein loading. After being washed with TBST thrice, membranes were incubated with horseradish peroxidase- (HRP-) conjugated IgG (1 : 40000, Jackson Lab) for 1 hour at RT. Blots were then developed with enhanced chemiluminescence developing solutions and quantified.

### 2.11. Enzyme-Linked Immunosorbent Assay (ELISA)

Caspase-3 activity in AML cells from different groups was assayed using Quantikine caspase-3 (R&D System) as previously reported [17]. In brief, AML cells were harvested, washed in PBS, and resuspended in extraction buffer containing protease inhibitors. Standard and sample extracts were added to the microplate precoated with antibody specific for caspase-3. HRP substrate was added to each well. The level of caspase-3 was measured at 450 nm.

### 2.12. Statistical Analysis

Statistical analysis was done using SPSS software version 16.0. All experiments were carried out at least three times. Data are shown as mean ± SEM, unless otherwise noted. Comparison for two groups was examined by Student's* t*-test. Multiple comparisons were performed by one-way ANOVA. For all cases, statistical significance was set as *P* < 0.05.

## 3. Results

### 3.1. Characterization of LSCs

**Figure 1:**
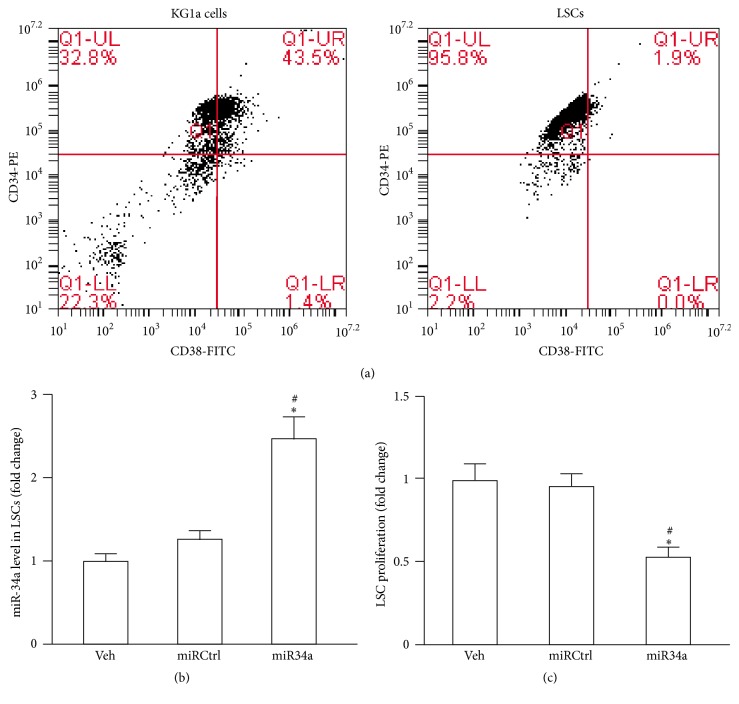
LSC characterization and the effect of miR34a overexpression on LSC proliferation. (a) Representative flow cytometric plots of CD34-PE versus CD38-FITC. Expressions of CD34 and CD38 in KG1a cells before sorting (left) and after sorting (right). (b) LSCs were subjected to vehicle (veh) treatment and transfected with miRCtrl or miR34a mimic as indicated and 2 days later the miR34a level was measured by qRT-PCR. (c) LSCs were subjected to treatment with veh or miRCtrl transfection or miR34a mimic transfection. After 2 days, the LSC proliferation was determined by MTT assay. Data represents mean ± SEM. ^*∗*^ *P* < 0.05 versus veh; ^#^ *P* < 0.05 versus miRCtrl.

To determine the proportion of LSCs in KG1a cells and whether LSCs were successfully isolated from KG1a cells, KG1a cells and microbeads-isolated LSCs were stained with CD34-PE and CD38-FITC antibodies [18, 19] and analyzed by flow cytometry. As shown in Figure 1(a), the KG1a cells contained about 32.8% of CD34+CD38− cells. After microbead sorting procedure, more than 95% of the cells were CD34+CD38− and considered LSCs.

### 3.2. The Effect of miR34a Overexpression on LSC Proliferation

The transfection efficiency of miR34a mimic was evaluated by qRT-PCR (Figure 1(b)). As expected, the level of miR34a in LSCs was significantly elevated after miR34a mimic transfection when compared with miRCtrl transfection or veh. This finding indicates successful overexpression of miR34a in LSCs. Overexpression of miR34a remarkably inhibited the proliferation ability of LSCs (versus control, *P* < 0.05; versus miRCtrl, *P* < 0.05; Figure 1(c)).

### 3.3. The Particle Concentration and miR34a Levels of LMVs, LMV^miRCtrl^, and LMVs^miR34a^

**Figure 2:**
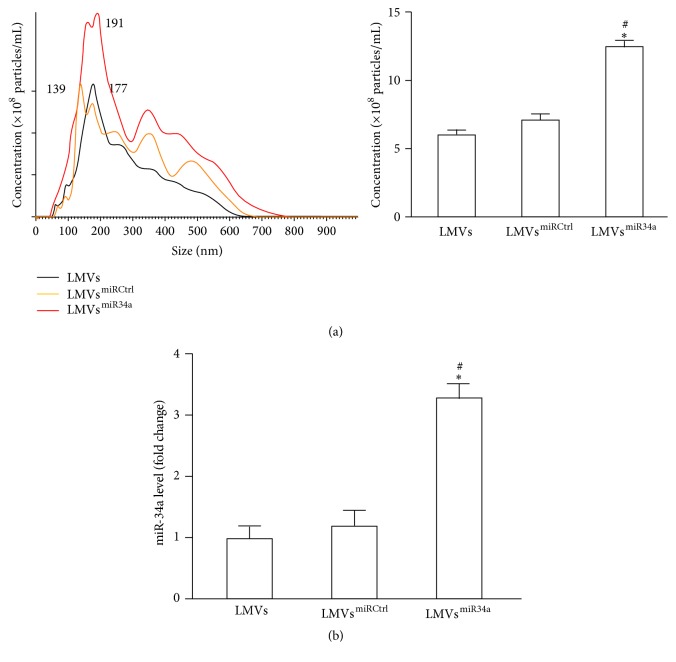
The particle number and miR-34a level of LMVs, LMVs^miRCtrl^, and LMVs^miR34a^. (a) Representative NTA plots of MV size distribution (left) and concentration (right). (b) qRT-PCR analysis of miR-34a level in LMVs, LMVs^miRCtrl^, and LMVs^miR34a^. Data represents mean ± SEM. ^*∗*^ *P* < 0.05 versus LMVs; ^#^ *P* < 0.05 versus LMVs^miRCtrl^. LMVs represent MVs generated from LSCs without transfection; LMVs^miRCtrl^ represent MVs generated from LSCs transfected with miRCtrl; and LMVs^miR34a^ represent MVs generated from LSCs transfected with miR34a mimic.

As shown in Figure 2(a), NTA analysis showed that the number of LMVs^miR34^ was higher than that of LMVs or LMVs^miRCtrl^, although there were no significant changes in their size distribution. More interestingly, the expression of miR34a in LMVs was displayed in the same pattern as their parent LSCs. As shown in Figure 2(a), qRT-PCR assay showed that the level of miR34a in LMVs^miR34^ was much higher than that of LMVs or LMVs^miRCtrl^. There was no significant difference of miR34a level between LMVs and LMVs^miRCtrl^.

### 3.4. The Effects of LMVs^miRCtrl^ and LMVs^miR34a^ on Apoptosis, Proliferation, and Migration of AML Cells

**Figure 3:**
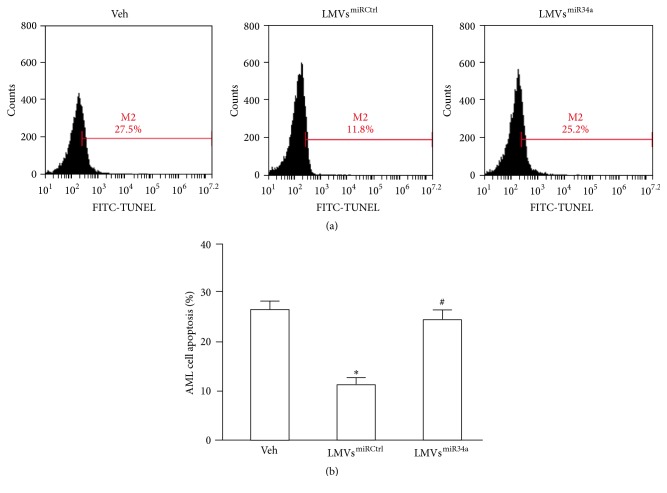
Effects of LMVs^miRCtrl^ and LMVs^miR34a^ on AML cell apoptosis. (a) Representative flow cytometric plots of AML cell apoptosis (percentage of FITC-TUNEL positive cells) after coincubation with veh, LMVs^miRCtrl^, or LMVs^miR34a^. (b) Summarized data of AML cell apoptosis. Data represents mean ± SEM. ^*∗*^ *P* < 0.05 versus veh; ^#^ *P* < 0.05 versus LMVs^miRCtrl^. Veh: fresh culture medium; LMVs^miRCtrl^: LMVs generated from miRCtrl transfected LSCs; and LMVs^miR34a^: LMVs generated from miR34a mimic transfected LSCs.

**Figure 4:**
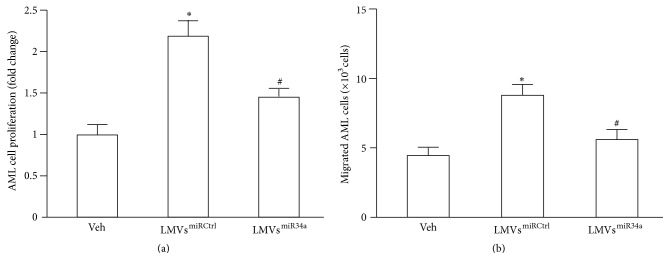
Effects of LMVs^miRCtrl^ and LMVs^miR34a^ on AML cell proliferation and migration. (a) Summarized data of AML cell proliferation which was determined by MTT assay. (b) Summarized data of AML cell migration which was determined by transwell assay. Data represents mean ± SEM. ^*∗*^ *P* < 0.05 versus veh; ^#^ *P* < 0.05 versus LMVs^miRCtrl^. Veh: fresh culture medium; LMVs^miRCtrl^: LMVs generated from miRCtrl transfected LSCs; and LMVs^miR34a^: LMVs generated from miR34a mimic transfected LSCs.

AML cell apoptosis (Figure 3) was identified by TUNEL staining and analyzed by flow cytometry. The apoptosis in AML cells was decreased after coincubation with LMVs^miRCtrl^ when compared with veh or LMVs^miR34a^ (11.29 ± 1.59% versus 26.70 ± 1.90%, *P* < 0.05 versus veh; 11.29 ± 1.59% versus 24.53 ± 2.30%, *P* < 0.05 versus LMVs^miR34a^; and *n* = 3). There was no significant difference in apoptosis between veh-treated and LMVs^miR34a^-treated AML cells (26.70 ± 1.90% versus 24.53 ± 2.30%, *P* = 0.63; *n* = 3). Moreover, the proliferation (Figure 4(a)) and migration abilities (Figure 4(b)) of AML cells were enhanced after coincubation with LMVs^miRCtrl^ (*P* < 0.05 versus veh; *n* = 3) whereas these effects were absent in AML cells coincubated with LMVs^miR34a^ (*P* > 0.05 versus veh; *n* = 3).

### 3.5. The Effects of LMVs^miRCtrl^ and LMVs^miR34a^ on Caspase-3 Activity and Tim-3 Expression in AML Cells

**Figure 5:**
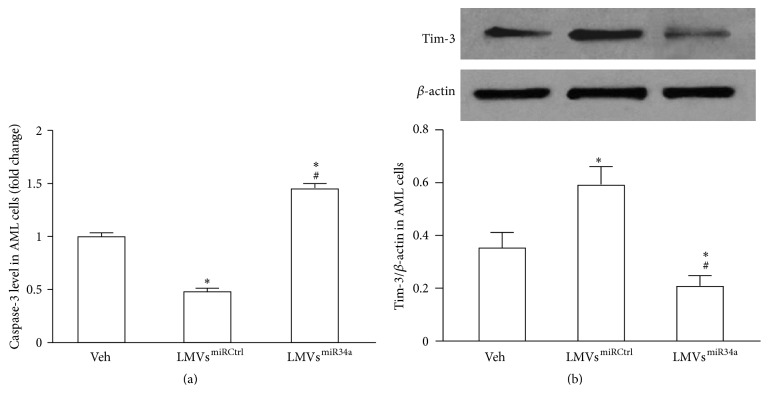
Effects of LMVs^miRCtrl^ and LMVs^miR34a^ on the levels of caspase-3 and Tim-3 protein in AML cells. (a) The activity of caspase-3 in AML cells which was determined by ELISA. (b) The expression of Tim-3 protein in AML cells which was determined by western blot. Data represents mean ± SEM. ^*∗*^ *P* < 0.05 versus veh; ^#^ *P* < 0.05 versus LMVs^miRCtrl^. Veh: fresh culture medium; LMVs^miRCtrl^: LMVs generated from miRCtrl transfected LSCs; and LMVs^miR34a^: LMVs generated from miR34a mimic transfected LSCs.

The activity of caspase-3 was reduced in AML cells coincubated with LMVs^miRCtrl^ (*P* < 0.05 versus veh; Figure 5(a)). LMVs^miR34a^ significantly increased the activity of caspase-3 in AML cells (*P* < 0.05 versus veh; Figure 5(a)). The expression of Tim-3 was upregulated in LMVs^miRCtrl^-treated AML cells (*P* < 0.05 versus veh; Figure 5(b)) but was downregulated in LMVs^miR34a^-treated AML cells (*P* < 0.05 versus veh; Figure 5(b)).

## 4. Discussion

There are several major findings in this study. Firstly, we demonstrated that LMVs promote the proliferation, migration, and survival of AML cells, which is associated with miR34a deficit since overexpression of miR34a in LSCs inhibited their proliferation. Secondly, LSCs with miR34a mimic transfection generate LMVs containing high level of miR34a (e.g., LMVs^miR34a^), which exert opposite effects on AML cells as compared to LMVs. Thirdly, the different effects of LMVs and LMVs^miR34a^ on AML cells were associated with their ability to differently regulate miR34a-targeted caspase-3 and Tim-3 pathways.

The concept of cancer stem cells has been proposed for many years and received increasing attention in recent years. There is solid evidence that AML contains a subpopulation of LSCs that are largely responsible for the refractory and resistance of AML to chemotherapy and immunotherapy [18, 20, 21]. However, the underlying mechanisms have not been fully understood. In our study, we isolated the CD34+CD38− fraction (LSCs) from KG1a cells by using the immune-magnetic microbead method as previously reported [18] and cultured for generating MVs. Elevated level of MVs has been found in various diseases including cancer, suggesting that they may serve as diagnostic and prognostic tool [22]. In addition, MVs can act as autocrine or paracrine mediators since they can merge with target cells to exert a wide range of actions [13] whereas whether LMVs have effects on the survival and malignance of AML remains unknown. In our study, we discovered for the first time that LMVs can promote the proliferative and migrative abilities and prevent the apoptosis of AML cells. A recent study has demonstrated that MVs derived from renal cancer stem cells induced abnormal endothelial cell growth, invasion of matrix, and resistance to apoptosis [6]. Therefore, our findings provide novel insight into previous perspectives [5, 6], suggesting that MVs derived from cancer stem cells can change the phenotypes of surrounding tumor and stromal cells to create a favorable tumor microenvironment.

Next, we examined the potential mechanisms of LMVs-induced effects on AML cells. It has been recently proposed that cancer stem cells not only initiate cancers but also support the progression of cancer in virtue of their oncogenic contents [23]. MVs are considered ideal vehicles for transferring the contents of cancer stem cells to bystander cells. Previous studies have also suggested that the RNA contents (mainly miRs and mRNAs) of MVs play a critical role, since the RNase treatment of MVs significantly inhibited the biological effects of MVs [6]. We therefore hypothesize that reprogramming the miR contents of LMVs could be able to alter the effects of LMVs on AML cells. Tumor-suppressive miR34a has been reported to be downregulated in most cancers, and restoration of miR34a has been shown to induce apoptosis and enhance chemosensitivity of cancer cells [24, 25]. Our current study found that overexpression of miR34a in LSCs not only significantly inhibited their proliferative ability but also produced LMVs containing high level of miR34a which could inhibit the effects of LMVs on AML cells. These data suggest a potential role of miR34a in the maintenance of LSCs and in LMVs-mediated effects, which may offer a novel way to target LSCs as well as modulate the interaction between LSCs and AML cells.

The downstream target genes of miR34a such as caspase-3 and Tim-3 have been identified in AML [9–12]. As the key effector of cellular death, caspase-3 activity is a predictor of survival in AML [9, 10]. A previous study has reported that overexpression of miR34a significantly induced pancreatic *β* cell apoptosis accompanied with increased caspase-3 activity [26]. In our study, we found that LMVs could significantly decrease caspase-3 activity in AML cells, which was inhibited by miR34a overexpression. Tim-3, highly expressed in human AML cells, can be served as a promising candidate for AML therapy [11, 12]. A previous report has shown that the high expression of Tim-3 is correlated with higher metastatic potential and shorter overall survival of cervical cancer [27]. Search for miR targets by using TargetScan confirmed that Tim-3 has a putative miR34a binding site within its 3′-UTR. We found that LMVs significantly upregulated Tim-3 protein expression in AML cells and these effects were reversed by LMVs carrying high level of miR34a. However, we did not directly test whether miR34a can target Tim-3 mRNA by binding to its 3′UTR and investigated whether overexpression of Tim-3 protein could rescue the effects of miR34a overexpression at this time. We acknowledge that there are some limitations in our study and it would be necessary to incorporate these questions into our future work. Of particular note, miR34a overexpression could partially block the effects of LMVs on AML cell proliferation, migration, and proliferation, although it was able to significantly elevate caspase-3 activity and decrease Tim-3 expression in AML cells; these data suggests that other miRs and associated mechanisms may also participate in LMVs-induced effects. Altogether, the involvements of miR34a in LMVs and its downstream targets, caspase-3 and Tim-3, in the malignance of AML were demonstrated in our study. Nevertheless, the detailed mechanisms of the roles of LMVs may need further investigation.

## 5. Conclusion

Our data demonstrate that LMVs are able to promote the proliferation and migration and inhibited apoptosis of AML cells, which is associated with the deficit of miR34a. Restoration of miR34a not only inhibits the LSC proliferation but also inhibits these effects of LMVs on AML cells via modulating caspase-3 and Tim-3 levels. These findings indicate that LMVs support the malignancy of AML cells and targeting of miR34a in LMVs could offer a novel approach for treatment of AML.
